# Instability of Water Quality of a Shallow, Polymictic, Flow-Through Lake

**DOI:** 10.1007/s11270-018-3724-2

**Published:** 2018-04-18

**Authors:** Beata Ferencz, Jarosław Dawidek, Magdalena Toporowska

**Affiliations:** 1 Department of Hydrobiology and Protection of Ecosystems, University of Life Sciences, 13Akademicka St, 20-950 Lublin, Poland; 2 Department of Hydrology, Maria Curie-Skłodowska University, Aleja Kraśnicka 2 cd, 20-718 Lublin, Poland

**Keywords:** Polymictic lake, Water quality, Nutrient retention, Trophy shift

## Abstract

This paper describes catchment processes that favor the trophic instability of a shallow polymictic lake, in which a shift from eutrophy to hypertrophy occurs rapidly. In the lake, in 2007, the winter discharge maximum and an intensive precipitation (monthly sums exceeded 60 mm) in a vegetation season were observed. In 2007, the cyanobacterial blooms disappeared and the water trophy decreased. Total phosphorus (TP) was the main factor determining the high trophic status of the lake. The TP retention resulted from a quick flow of two inflows: QI1 (r = 0.64) and QI2 (0.56), and the base flow of tributary 1 (0.62). A significant negative correlation between TP and precipitation (*r* = − 0.54) was observed. Both the surface and the groundwater inflow of I4 showed a positive correlation with the retention of PO_4_ (*r* = 0.67 and *r* = 0.60, respectively), whereas the outlet discharge determined RNO_3_ (*r* = 0.57). The trophy of Lake Syczyńskie was determined by the relationship between nutrient input and export, expressed as the ionic retention, Carlson’s trophic state index (TSI), and phytoplankton abundance. The results showed that many factors influence the stability of water quality in small, polymictic lakes. However, in the studied lake, intense precipitation and winter discharge maxima (particularly base flow) prevented summer cyanobacterial blooms.

## Introduction

Shallow water ecosystems are widespread worldwide. Similar to the other water bodies, they are a very important source for irrigation, cooling, recreation, and dilution of wastes and pollutants (Wetzel 2001). Meerhoff and Jeppesen (2009) define shallow lakes as more or less permanent standing water bodies, whose low depth allows light to penetrate to the lake’s bottom. They are usually polymictic in nature, and the water column is often intensively mixed even in summer. Scheffer et al. (1993) divided shallow temperate lakes into two categories in terms of their alternative equilibrium state: a clear state dominated by aquatic vegetation, and a turbid state, in which phytoplankton blooms occur. The state of a lake is highly dependent on the nutrient concentration. De-eutrophication (change back to a clear, macrophyte-dominated state) as a result of a reduction of the external load is often delayed by stabilizing mechanisms that cause resilience (Hilt et al. 2006). Lakes are not isolated from the surrounding area—terrestrial catchment; thus, they receive nutrient loading, resulting from the condition of the catchment (Kebede et al. 2006; Ito et al. 2009; Schauser and Chorus 2009). Fluctuations of nutrient loading are usually linked to human pressure; however, short-term variations in weather and runoff may influence the nutrient input from the external sources (Romarheim et al. 2015). Runoff caused by intense precipitation or snow melting affect water turbidity, hence also eutrophication (Jeppesen et al. 2009). As the nutrient load from the terrestrial catchment is significantly higher than that due to atmospheric precipitation (Ludovisi and Gaino 2010), a nutrient budget of lakes is often constructed assuming that a majority of the nutrients enter a lake through its tributary. Nitrogen and phosphorus are the most important factors limiting algal growth in lakes and thus, influence eutrophication processes (Conley et al. 2009; Smith and Schindler 2009). Nitrogen is supplied from the groundwater and runoff and through atmospheric deposition (Lampert and Sommer 2007; Koch et al. 2014), while biologically available phosphorus is supplied from the lake catchment (Tõnno et al. 2013). Degradation of water quality may result in a loss of ecological functions and a degradation of aquatic ecosystems (European Parliament and Council 2000; Akdeniz et al. 2011; Raicevic et al. 2011). It not only determines the changes in the species composition but also leads to the proliferation of toxic cyanobacterial blooms (Paerl et al. 2014).

The aim of the present study was to determine the external causes of the rapid shift between hypertrophy and eutrophy in a small, shallow Polish lake. The hypothesis were that (a) the de-eutrophication processes resulted from complex relationships between the allochthonous matter delivered from the lake catchment and (b) the hydrological factors, e.g., water residence time, favored the shift of the trophic state in the studied lake.

## Materials and Methods

### Study Area

**Fig. 1:**
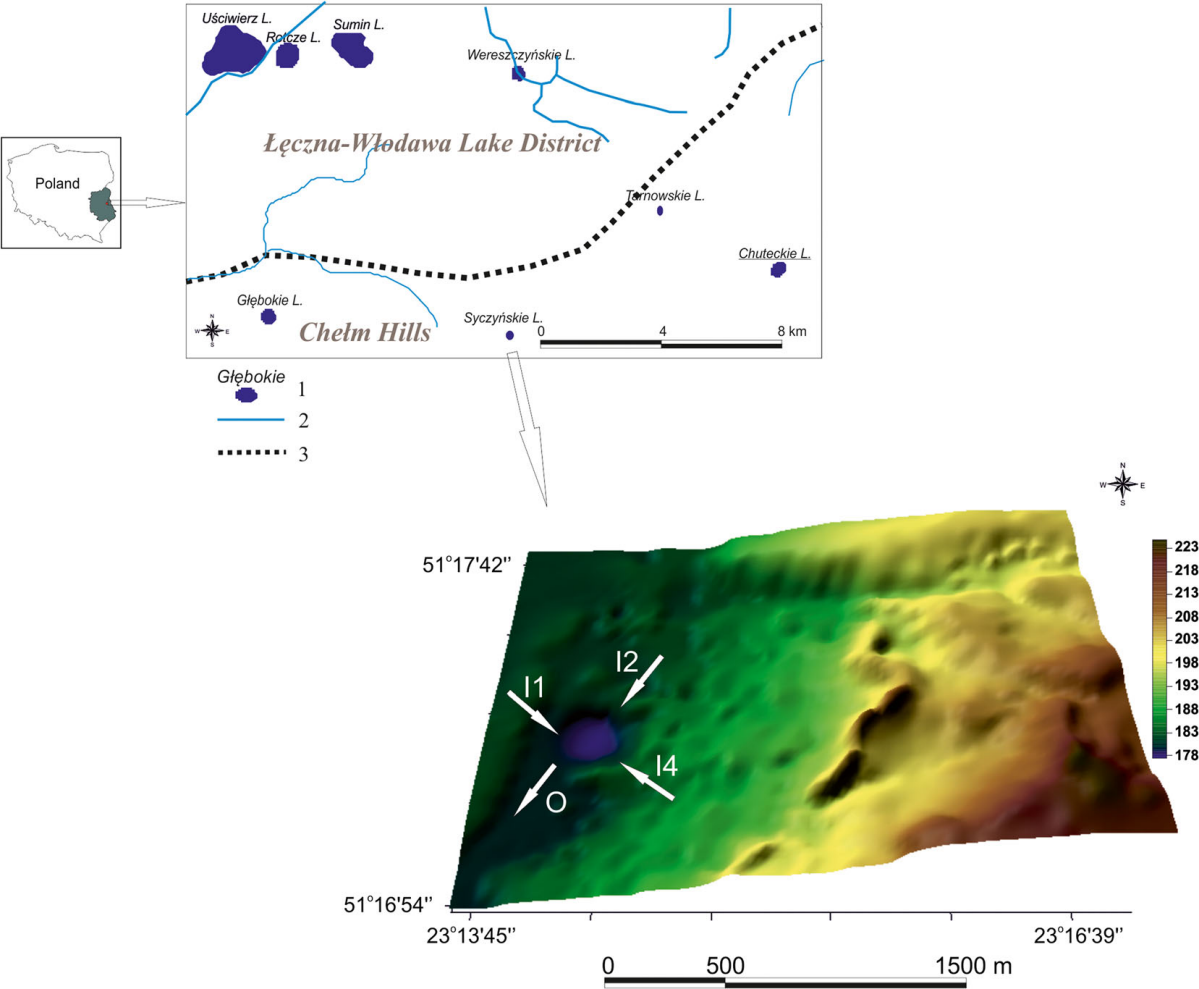
Location of Lake Syczyńskie (1) lakes, (2) rivers, (3) mesoregion’s border, (I1-I4) lake tributaries, (O) lake outlet

Lake Syczyńskie is a small (7.4 ha) and shallow (3.0 m maximum depth) polymictic water body, located in the boundary zone between Lublin Upland (Chełm Hills) and Polish Lowlands (Łęczna-Włodawa Lake District). The geographical coordinates of the lake’s deepest part are as follows: 51° 17′ 12″ N latitude and 23° 14′ 17″ E longitude. The lake basin is fed by the waters of four tributaries (Fig. 1): one perennial (I2), two intermittent (I1 and I4), and one episodic (I3), which has been excluded from the analysis. The lake is drained to the southwest, into the Świnka River. The lake is considered hypertrophic, and cyanobacterial blooms have been observed since 2000 (Kornijów and Pęczuła 2005). A distinct domination of cyanobacteria, which accounted for 74–94% of the total phytoplankton abundance with the predomination of filamentous *Planktothrix aghardii* (Gom.) Anagn & Kom, was observed in the lake (Pęczuła et al. 2014).

### Hydrological Measurements

The water levels of both the lake and the streams were observed daily. The flow rates were measured bimonthly by using an OTT Nautilus 2000 flow meter. Water samples for the chemical analyses were collected once a month, 1 m below the surface from the deepest part of the lake and at 0.8 of the maximum depth of the current zone from the streams that supplied and drained the Lake Syczyńskie basin. In addition, daily sums of precipitation were observed using Hellman’s pluviometer. The flushing time was calculated after Rueda et al. (2006) using the following equation:

$$ Tf=V\kern0.5em \ast \kern0.5em {Q}^{-1} $$

where Tf is the flushing time, *Q* is the daily underground output, and *V* is the daily lake volume.

The lake’s supply of dissolved mineral solids was characterized as the hydrochemical potential of sub-catchments and presented as its ionic specific load. The specific load [kg year^−1^] was calculated by multiplying the discharge and the ion concentration, after Cai et al. (2012).

Chemical analyses were performed using a Slandi LF300 photometer. The concentrations of NO_3_, NH_4_, PO_4_, and total phosphorus (TP) were measured in samples collected once a month from the deepest part of the lake, inlets, and outlet. Samples were collected using 250-mL flasks and then, transported to the laboratory in a cooler. The detection range of the ions was 0.1–30 mg L^−1^ for NO_3_, 0.01–2.0 mg L^−1^ for NO_2_, 0.01–5.0 mg L^−1^ for PO_4_, and 0.02–1.1 mg L^−1^ for TP. The spectral range was 520 nm for NO_3_ and NO_2_ and 700 nm for PO_4_ and TP.

Samples were preserved using sulfuric acid to determine the TP concentration. In the laboratory, the samples were mineralized in the microwave oven for 30 min before the measurements.

The nutrient retention was calculated after Mosello et al. (2001) as follows:

$$ Rx=\frac{Lx_{in}-{Lx}_{out}}{Lx_{in}} $$

where *Rx* denotes the retention of an ion *x*, *Lx*_*in*_ indicates the input loading rate, and *Lx*_*out*_ represents the output loading rate.

### Trophic Status of the Lake and Cyanobacterial Blooms

Carlson’s trophic state index (TSI) based on the water transparency (measured in situ with a Secchi disk [SD]), concentrations of TP, and chlorophyll-*a* (determined spectrophotometrically; PN-ISO, 10260 2002, Specord 40 Analytik Jena) were calculated according to Carlson’s formulas (1977). Carlson (1977) recommended basing the trophic state classification of lakes on the data from summer. However, we used TSI to analyze the variability of the trophic state of Lake Syczyńskie among seasons and years according to the method proposed by Gołdyn et al. (2003), Deng et al. (2014), and Liu et al. (2017). The abundance of phytoplankton (prokaryotic cyanobacteria and eukaryotic algae) was analyzed mostly based on Toporowska and Pawlik-Skowrońska (2014) and, in the case of four research terms, on Toporowska (2011).

### Statistical Analyses

Ordination techniques were used to demonstrate the hydrometeorological relationships: quick flow of the inlets (QI1, QI2, and QI4), base flow of the inlets (BI1, BI2, and BI4), as well as quick flow and base flow of the outflow (QO and BO), precipitation (P), flushing time (Tf), and the chemical ones, calculated as the ionic retention. An indirect multivariate detrended correspondence analysis DCA method was used to calculate the gradient indicated by the chemical variables. The length of the gradient allowed us to use an RDA analysis to determine the strongest relationships among the data. For the automatic forward selection of hydrometeorological variables, we performed a Monte Carlo permutation test (999 permutations) to determine the most important variables (significance must have exceeded 0.05).

## Results

### Hydrometeorological Conditions

**Fig. 2:**
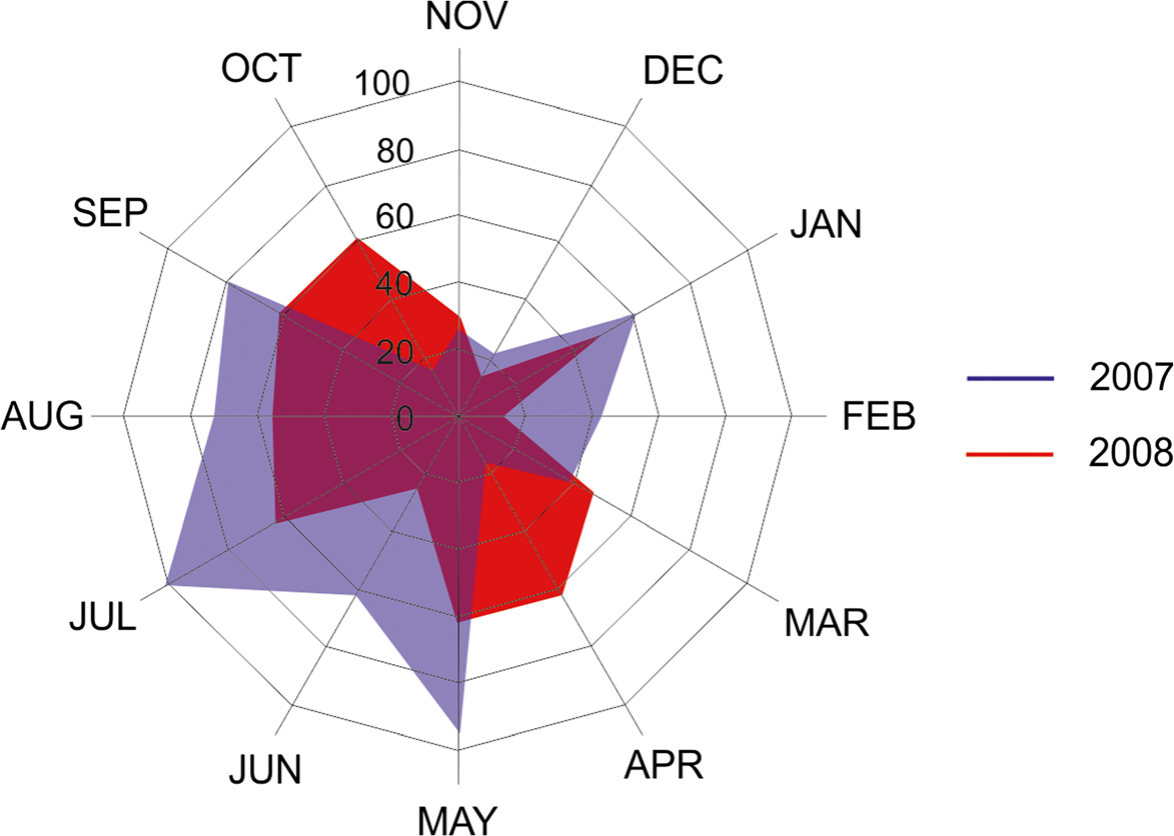
Monthly sums of precipitation [mm] during the study period (1) 2007 water year, (2) 2008 water year

Water years 2007–2008 (starting in November 2006 and ending in October 2008) varied in terms of the atmospheric supply (Fig. 2). In 2007, a higher annual sum of precipitation was observed than in 2008. The first year of study was characterized by the high volatility of monthly precipitation, from 16 mm in April to 101 mm in July. The entire growing season, in which in the previous years, the toxic blooms of cyanobacteria were observed, was characterized by a predominance of rainfall more than 60 mm (June to September). In 2008, a relatively high precipitation was recorded only from July to October.

### Hydrological Conditions

The flow-through Lake Syczyńskie was hydrologically highly active during the study period, which was visible in the high discharge values. In 2007, the discharge maxima of the main tributaries I2 and I4 were observed in winter (February and March), and several periods of increasing flow rates were observed in summer and autumn. In 2008, the highest discharges were observed in January and April, and the second peak was determined by the intensive precipitation in June. Winter thaws in 2007 favored a high surface runoff and the renewal of the lake water resources with low mineralized water. I2 was the most active tributary in the study period; its mean discharge amounted to 10.5 dm s^−1^. The discharge of streams I4 and I1 amounted to 6.4 and 5.0 dm s^−1^, respectively. The stability of the groundwater recharge, previously described by Dawidek and Ferencz (2014), was visible in the low ratio of the discharge irregularity (maximum/minimum value) of the I2 and I4 streams: 7.1 and 11.6, respectively.

**Fig. 3:**
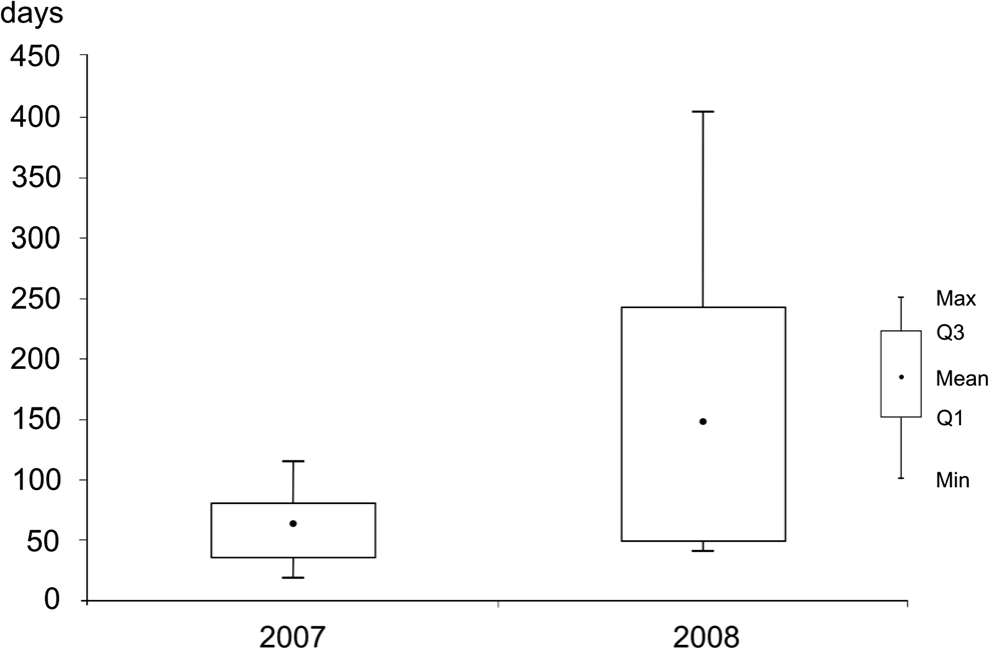
Variability of Tf in the study period

Although the flushing time was determined by the hydrological activity of the streams, the water years 2007 and 2008 varied considerably in terms of the flushing time. The interquartile range of the values in 2007 was smaller than that in 2008 (Fig. 3). The shortest lake water removal, observed in 2007 (19 days in August), was twice faster than that in 2008 (41 days in April). The mean values also showed that in 2007, Lake Syczyńskie was more intensely flushed than in 2008 (64 and 148 days, respectively). In both these years, the fastest flushing occurred in spring, from February to April. In 2008, an increase in the flushing rate was observed from June to October (exceeding 100 days).

### Hydrochemical Conditions

**Fig. 4:**
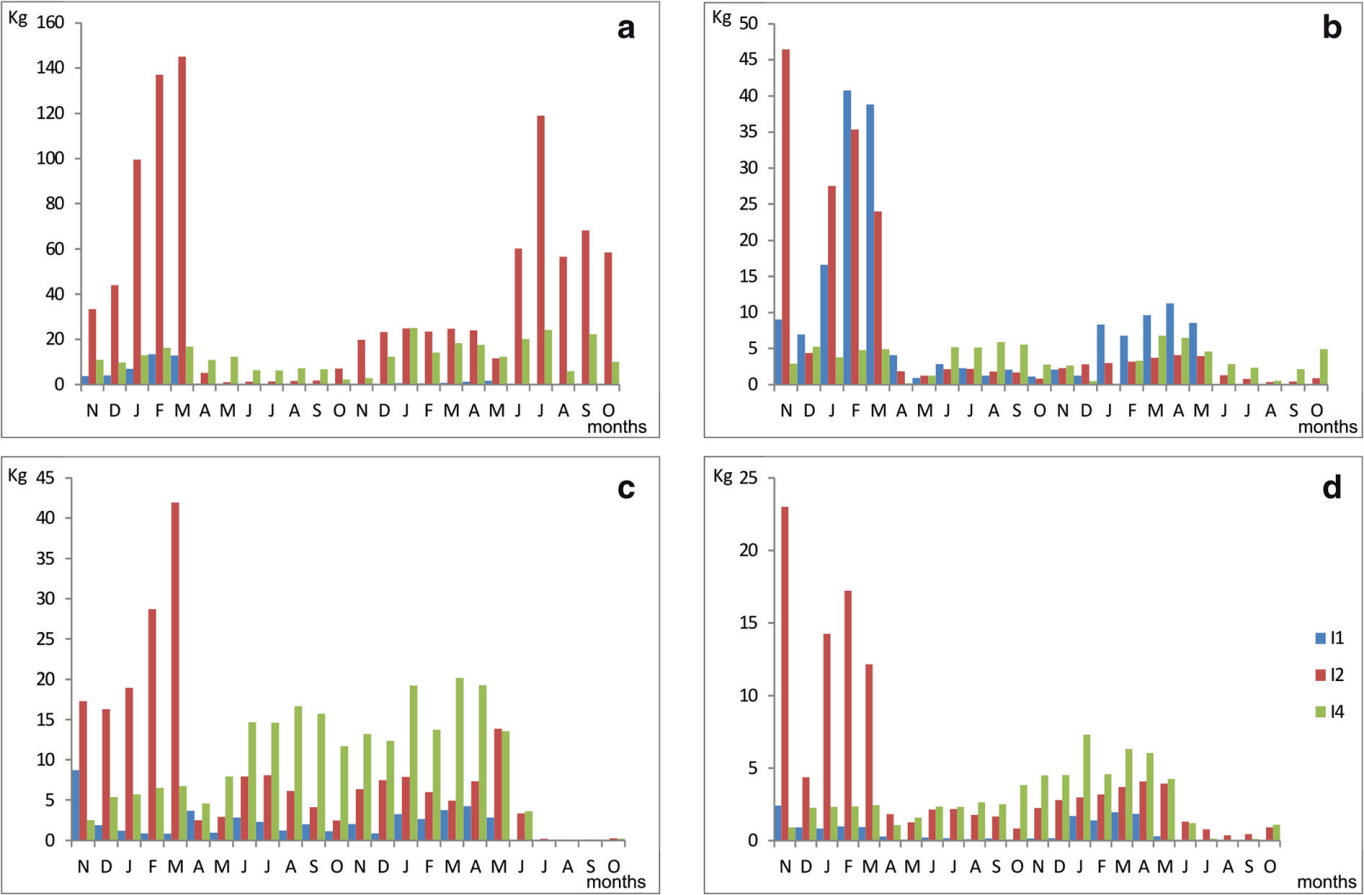
Variability of ionic load [kg year^−1^] in the study period. **a** NO_3_. **b** NH_4_. **c** PO_4_. **d** TP. I1, inflow 1; I2, inflow 2; I4, inflow 4

The monthly ionic load of the three main tributaries of Lake Syczyńskie is presented in Fig. 4. Among all of the ions, the streams carried the higher load of NO_3_ into the lake basin in the study period. The winter loads entering the lake in 2007 were several times higher than those in 2008. The opposite situation occurred in a warm season (Fig. 4). The highest load was delivered by the stream I2, which drained the Cretaceous hill slopes of the Chełm Hills. The agricultural catchment also released the high load of NH_4_. In this instance, elevated loads were also observed in the winter months, in both the study years. NH_4_ was mostly transported by stream I1, whose catchment was abundant with piles of cow manure. Although a similar interannual variability was observed in both the study years, the load of NH_4_ in 2007 was four to five times higher than that in 2008. The highest monthly load of PO_4_ exceeded 40 kg in the I2 stream in 2007. The highest values were observed in winter 2007 in the waters of the I2 tributary. From April 2007 to April 2008, a significant prevalence of the I4 stream in the ion transport was observed. The TP load showed the highest variability in stream I2, where the monthly loads in 2007 were always higher than those in the corresponding months of 2008. Stream I4 carried a higher load of TP in 2008.

The mass of NO_3_ ions entering the lake basin each month was higher than the load leaving the lake basin. Significant changes between the two study years were observed. In 2007, the highest NO_3_ retention values were observed in winter, from November to March (Table 1), whereas in 2008, two peaks of retention values were noted, the first in winter, and the second (higher) in the warm season (from June to October). The retention of NH_4_ showed high variability in the study period. In 2007, no seasonality was observed. However, except in the cold months (November, January, and February), a higher NH_4_ load was carried outside the catchment than that delivered to the lake basin (Table 1). A clear duality occurred in the water year 2008, when from the beginning of the year (November) to April, a negative ionic retention was observed, and from May to October, the ions accumulated in the lake waters. Differences between retention during both the study years were also clear for PO_4_ and TP. A similar fluctuation of the retention values occurred in 2008, whereas no similarity was observed in 2007. In 2007, a prevalence of the negative retention values of PO_4_ was observed, in contrast to 2008, when the ions accumulated in the lake waters. The retention of TP was positive in a majority of the cold months of 2007, whereas negative from April to September. In 2008, the supremacy of the ion input was observed (Table 1).

**Table 1:** Values of ionic retention calculated for the study period

Date	RNH_4_	RNO_3_	RPO_4_	RTP
Nov 2006	0.64	0.89	− 3.17	0.64
Dec 2006	− 1.12	0.67	− 6.45	− 0.66
Jan 2007	0.25	0.78	4.38	0.37
Feb 2007	0.01	0.64	1.12	0.08
Mar 2007	− 0.36	0.60	5.91	− 0.43
Apr 2007	− 1.94	0.85	− 44.61	− 1.61
May 2007	− 1.11	0.90	− 9.13	− 0.53
Jun 2007	− 0.42	0.17	− 5.60	− 1.45
Jul 2007	− 0.54	0.14	− 6.84	− 1.51
Aug 2007	− 0.07	0.51	3.90	− 0.68
Sep 2007	− 0.69	0.33	− 10.73	− 1.77
Oct 2007	− 1.37	0.71	5.90	0.13
Nov 2007	− 0.42	0.92	19.87	0.72
Dec 2007	− 2.79	0.93	28.05	0.77
Jan 2008	− 0.97	0.93	51.26	0.81
Feb 2007	− 2.05	0.79	18.43	0.36
Mar 2008	− 1.18	0.80	23.73	0.44
Apr 2008	− 0.83	0.80	24.72	0.39
May 2008	0.49	0.79	11.19	0.09
Jun 2008	0.62	0.93	6.22	0.56
Jul 2008	0.82	0.97	2.40	0.91
Aug 2008	0.73	0.95	2.00	0.88
Sep 2008	0.85	0.95	3.64	0.91
O 2008	0.11	0.96	3.75	0.20

**Fig. 5:**
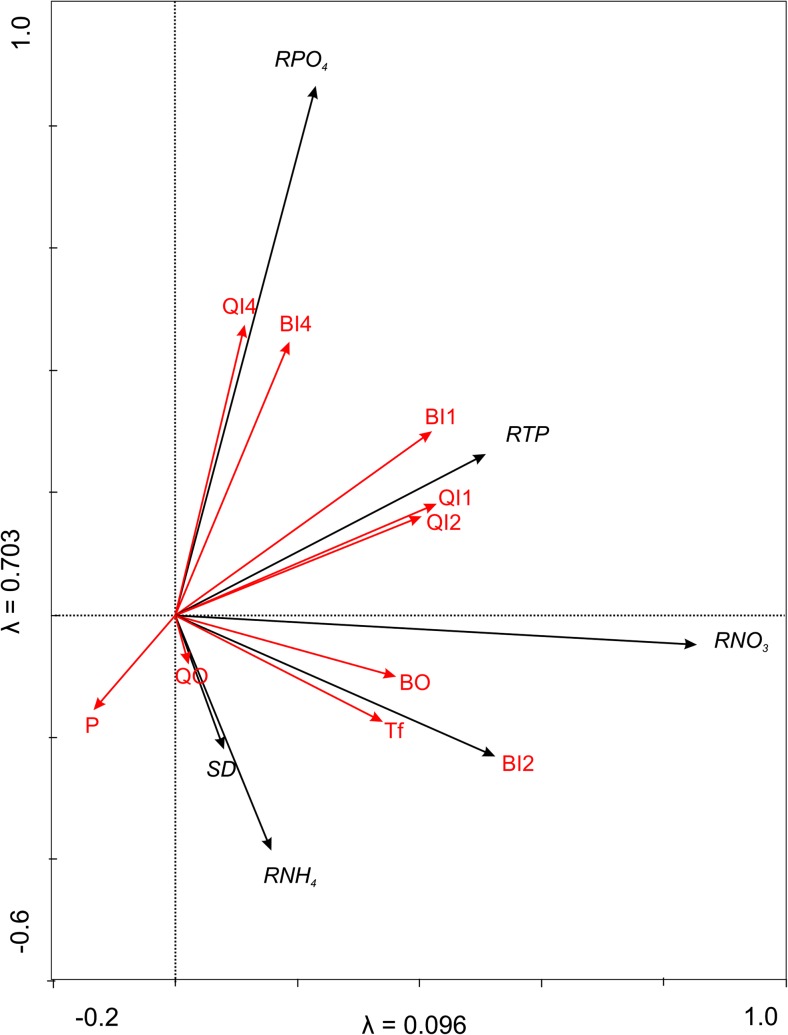
Redundancy analysis (RDA) biplot showing statistical relationships between hydro-meteorological factors and ionic retention value. I1–I4, inflows; O, outflow; BI1–BI4, base flow of the inflows; BO, base flow of the outflow; QI1–QI4, surface runoff of the inflows; QO, surface runoff of the outflow RNO_3_, RPO_4_, RNH_4_; RTP, ionic retention of the particular ion; Tf, flushing time; P, precipitation; SD, Secchi disc

Figure 5 shows the statistical relationships between the hydrometeorological factors and the ionic retention value. All of the chemical variables explained 82.2% of the total variance. However, the hydrometeorological variables that significantly explained the variance in the ionic retention were as follows: base flow of stream 2 (*λ* = 0.42; *F* = 16.19; *p* = 0.002), surface runoff of stream 1 (*λ* = 0.14; *F* = 10.05; *p* = 0.006), and Tf (*λ* = 0.18; *F* = 9.6; *p* = 0.004). In general, phosphorus showed a strong dependence on the hydrological factors. The retention value of TP was attributed to QI1 (*r* = 0.64), BI1 (*r* = 0.62), and QI2 (*r* = 0.56). However, it showed a negative correlation with the precipitation (*r* = −0.54). The retention of PO_4_ was determined by the groundwater (*r* = 0.60) and the surface runoff (*r* = 0.67) of tributary 4. The underground component of the outlet determined the retention of NO_3_ (*r* = 0.57) (Fig. 5).

### Lake Trophy and Phytoplankton Blooms

**Fig. 6:**
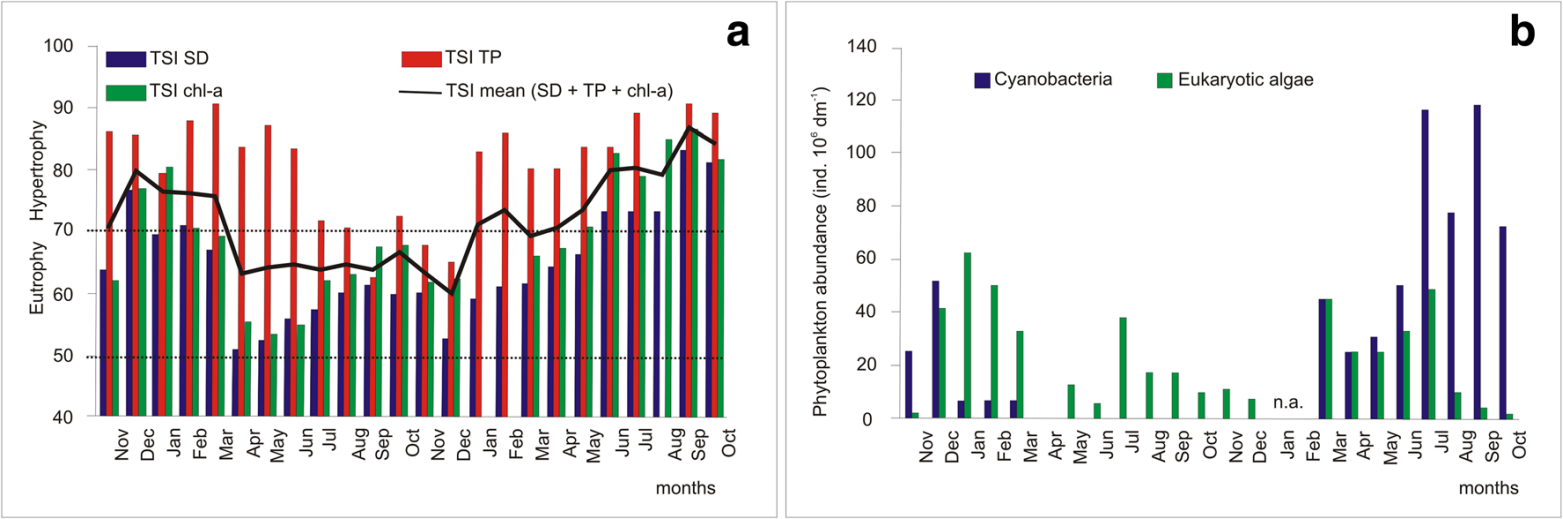
Changes in the trophic status (**a**) and development of cyanobacteria and other phytoplankton groups (**b**) in the studied period. n.a. not analyzed

TSI (Fig. 6a) reached the highest values, characteristic for the hypertrophy, in the beginning of the study period, from November 2006 to March 2007 (TSI mean_SD + TP + chl-*a*_ = 71–80), and in 2008, from January to October (69–87). In 2007, the TSI values decreased considerably, and from April to October, the TSI mean range was 63–67, whereas in December, it decreased up to 58, indicating the eutrophic character of the lake. The TP concentration was high during the entire study period (0.104–0.406 mg dm^−1^) except for two sampling occasions (< 100 mg dm^−1^) and was the most important factor responsible for the high trophic status of the lake, including 2007. Strong cyanobacterial blooms (Fig. 6b) were observed in the lake in November and December 2006 and in all of 2008, and were caused mainly by the filamentous species *P. agardhii*, which reached maximal abundance (2.1 × 10^7^–11.6 × 10^7^ ind. L^−1^) in the summer months (August and September) of 2008 (Toporowska and Pawlik-Skowrońska 2014). Other cyanobacteria such as *Planktolyngbya limnetica* (Lemm.) Kom.-Legn. & Cronberg, *Aphanizomenon gracile* (Lemm.), and *Limnothrix redekei* (Van Goor) Meffert reached high abundances seasonally. The decrease in the lake trophy observed in 2007 (Fig. 6a) was parallel with the disappearance of the cyanobacterial blooms; however, the abundance of eukaryotic algae was still high (up to 38 × 10^6^ ind. dm^−3^, Fig. 6b). Among the eukaryotes, some Bacillariophyceae predominated in the winter and late autumn seasons, whereas in spring and summer, Cryptophyceae and different types of Chlorophyceae were abundant (Toporowska 2011; Toporowska and Pawlik-Skowrońska 2014).

## Discussion

The distribution and intensity of rainfall is considered a factor leading to the changes in the physicochemical structure of the water bodies (Bouvy et al. 2003) and the phytoplankton communities. A large amount of precipitation may influence the water quality, leading to a dilution of the nutrients (Reichwaldt and Ghadonani 2012). Moreover, the high quantity of precipitation, particularly in small lakes, may lead to a reduction of the algal biomass because of the high flushing rates (Figueredo and Giani 2009; Kuhn et al. 2011). The flushing rates of the warm season May–September 2007, because of the precipitation quantity, amounted to circa 2 months. Thus, precipitation was a factor that favored a shift in Lake Syczyńskie’s trophy. The precipitation intensity in 2008 was low, and the cyanobacterial blooms were observed then.

The results confirmed that the hydrology of the inflows had a significant effect on the quality of the lake water and the cyanobacterial blooms, as the allochtonous nutrient input entered the lakes mostly via tributaries (Janssen et al. 2014). Further, the water flushing time was believed to be very important in shaping the average nutrient and chlorophyll concentrations in the lakes (Janse et al. 2010). Recently, Pawlik-Skowrońska and Toporowska (2016) reported that the increased flushing rate caused a decrease in the cyanobacterial blooms and the cyanotoxin production in two hydrologically transformed lakes in Eastern Poland. Both the maxima and the minima, as well as the mean values, proved that the water of the lake under study was intensely renewed in 2007. The flushing time of the second year of study was significantly longer.

The high input of NO_3_ was attributed to the agricultural character of the lake catchment. More than 80% of the catchment was used as arable land (Pęczuła et al. 2009). Typically, the NO_3_ load shows a sinusoidal pattern, with high values during winter, and low in summer (Buhvestova et al. 2011). This was observed only in the second year of the study. During the first year of study, a reverse pattern was observed. Ionic retention is a very important factor shaping water quality, as it results from the values of the ionic input and the load leaving the lake. The variability of the TP retention was in contrast to the results reported by Dillon and Molot (1996) and Søndergaard et al. (1999), who stated that the TP retention peaked during summer and was at its minimum in winter. The TP input was lower than the export in the first year of study. Thus, the phosphorus retention was the factor that caused the lack of cyanobacterial blooms in 2007. Although the TP retention is assumed to be related with Tf, particularly in lakes with the high flushing rate (Erlandsson et al. 2008), it has not been confirmed in Lake Syczyńskie. Brett and Benjamin (2008) associated this with the fact that short flushing allows less time for the nutrient removal processes to occur.

The correlation of TP with the discharges of the streams with catchments abundant in Cretaceous sediments was positive, and it was negative for precipitation. This confirmed that the high concentration of calcium carbonate favored phosphorus retention (Hartley et al. 1997). However, no relationship between the TP retention and Tf has been observed, although it has been stated in other temperate lakes (Søndergaard et al. 2003). The negative correlation between TP and precipitation was attributed to the fact that the electrical conductivity of rainwater in the Lubelszczyzna region was approximately 20 μS cm^−1^ (Chmiel 2002). While the concentration of nitrogen in the rainwater was rather significant (circa 3.2 mg L^−1^ of NO_3_), trace levels of phosphorus were observed (Malec and Borowski 2015).

Toporowska and Pawlik-Skowrońska (2014) explained the lack of cyanobacterial blooms in 2007 by the low ratio of DIN/DIP at that time. However, the low value was not a cause of the trophy shift, but like water chemistry, it was an effect of complex hydrometeorological processes. The trophy of Lake Syczynskie was not determined by the ionic upload from the lake catchment but rather by the relation upload—export, expressed by ionic retention. In particular, phosphorus retention (both PO_4_ and TP) was a key factor shaping the water trophy. Phosphorus speciation, however, showed a strong correlation with the hydrological factors. The trophy shift observed in Lake Sczyńskie was very beneficial to the lake ecosystem because of the disappearance of the cyanobacterial blooms and the considerable reduction in the production of cyanotoxins, including hepatotoxic microcystins and neurotoxic anatoxin-a (Toporowska and Pawlik-Skowrońska 2014).

## Conclusions

Understanding the catchment mechanisms, by recognizing the hydrological and hydrochemical processes, is essential for the rational management of water resources, both quantitative and qualitative. Research has shown that appropriate control of the abovementioned processes will allow to achieve and maintain an improvement in the quality of the lake water. In the case of Lake Syczyńskie, this goal can be achieved by, e.g., (a) forcing the annual total lake water renewal during winter (high input of low mineralized, thawing water), (b) knowing that I4 is the main stream responsible for the retention of TP in the lake and eliminating the point sources of pollution in this sub-catchment (which is easier and cheaper than the action in the entire lake basin), and (c) limiting the hydrological role of the I4 stream in summer (using weir).

The instability of the lake’s water quality was attributed to complex hydrometeorological factors. Precipitation, its frequency and quantity, favored an improvement in the lake’s water quality. Moreover, the winter maxima of the tributaries discharge played a major role in water renewal, which hindered the summer cyanobacterial blooms.
